# SpikingLab: modelling agents controlled by Spiking Neural Networks in Netlogo

**DOI:** 10.1007/s00521-016-2398-1

**Published:** 2016-06-07

**Authors:** Cristian Jimenez-Romero, Jeffrey Johnson

**Affiliations:** Design-Complexity Group, The Open University, Milton Keynes, MK7 6AA UK

**Keywords:** Spiking neurons, Neural networks, Agents, Modelling, Simulations, Artificial life, Artificial intelligence, Robots, Membrane potential, Neural circuit, Spike timing, Dependent plasticity, STDP, Neuro engineering

## Abstract

The scientific interest attracted by Spiking Neural Networks (SNN) has lead to the development of tools for the simulation and study of neuronal dynamics ranging from phenomenological models to the more sophisticated and biologically accurate Hodgkin-and-Huxley-based and multi-compartmental models. However, despite the multiple features offered by neural modelling tools, their integration with environments for the simulation of robots and agents can be challenging and time consuming. The implementation of artificial neural circuits to control robots generally involves the following tasks: (1) understanding the simulation tools, (2) creating the neural circuit in the neural simulator, (3) linking the simulated neural circuit with the environment of the agent and (4) programming the appropriate interface in the robot or agent to use the neural controller. The accomplishment of the above-mentioned tasks can be challenging, especially for undergraduate students or novice researchers. This paper presents an alternative tool which facilitates the simulation of simple SNN circuits using the multi-agent simulation and the programming environment Netlogo (educational software that simplifies the study and experimentation of complex systems). The engine proposed and implemented in Netlogo for the simulation of a functional model of SNN is a simplification of integrate and fire (I&F) models. The characteristics of the engine (including neuronal dynamics, STDP learning and synaptic delay) are demonstrated through the implementation of an agent representing an artificial insect controlled by a simple neural circuit. The setup of the experiment and its outcomes are described in this work.

## Introduction

Artificial Neural Networks of third generation known as Spiking Neural Networks (SNN) have been gaining the attention of the scientific community in different disciplines, including neuroscience, computer science, cognitive science, physics and mathematics. This has lead to the development of tools (e.g. GENESIS [1], NEURON [2], SNNAP [3]) for the simulation and study of neuronal dynamics ranging from phenomenological models to the more sophisticated and biologically accurate Hodgkin-and-Huxley-based [4] and multi-compartmental models [5]. These tools have allowed experimenting with complex neural dynamics, from purely computational and artificial to biological scenarios. However, despite the multiple features offered by neural modelling tools, their integration with environments for the simulation of robots and agents can be challenging and time consuming if the user is not familiar with the technicalities behind the tools.

There are different approaches that can be taken in order to simulate agents and robots controlled by SNN circuits, depending of the objectives and requirements of the investigation being carried out (including level of biological accuracy, required performance, types of interactions between agents and their environment). These approaches can be summarized as follows: (1) using software interfaces: between two (or more) software applications where one of them simulates the SNN models, while the other application is responsible for the simulation of the robot/agent virtual environment; (2) using high-level programming languages (e.g. C++, Java, Python) combined with the corresponding programming libraries for the SNN models implementation and simulation of the robot/agent virtual world; (3) integrated simulation environments: using standalone software applications or Suites (e.g. AI-SIMCOG [6], AnimatLab [7]) that support the implementation of SNN models into agents/robots while providing a simulated virtual world for experimentation.

The interface approach has the advantage that the features of different simulation tools can be combined (i.e. iqr [8] with arduino [9], Brian [10] and SimSpark [11]). However, the possibility of combining two different applications depends on the communication interfaces of each application. In terms of simplicity, this approach requires also good technical knowledge of the different simulation tools and the software and hardware interfaces to be used. As a second choice, high-level programming languages such as C++ and Python are supported by a large community of open-source and commercial developers which are steadily creating and maintaining software libraries including those for SNN modelling (Nemo [12], Nest [13]) and agents and robots simulations (Gazebo [14], Webots [15]). On the other hand, the use of programming languages with specialized libraries requires knowledge of their technicalities including the software framework, functions, object types and the programming language itself. As an alternative, integrated simulation environments offer all-in-one tools for designing, implementing an experimenting with SNN models and agents/robots in simulated or real scenarios. However, this approach also requires good knowledge of the used tool including its graphical user interface (GUI), embedded functions and programming languages or scripts. Moreover, the process of modelling and simulating is subject to the features offered by the integrated environment.

Implementing artificial neural circuits to control agents or robots generally represents a very challenging task, especially for those who lack extensive experience with complex programming and simulation tools. This is because of the several tasks necessary in order to implement sophisticated neural circuits able to control autonomous systems (i.e. agents and robots). Firstly, a clear understanding of the simulation tools is required. Secondly, the neural circuit must be created in the neural simulator. Thirdly, the simulated neural circuit must be connected with the simulation environment of the agent (or with the communication interfaces of the robot). Lastly, the appropriate interface in the agent or robot must be programmed to use the neural controller. In order to simplify such complex tasks, this paper presents an alternative tool which facilitates the simulation of simple SNN circuits and their application in agents using the multi-agent simulation and programming environment Netlogo [16], educational software that simplifies the study and experimentation of complex systems. This paper proposes an engine implemented in Netlogo, for the simulation of a functional model of SNN based on a simplified version of I&F models [17, 18]. The coding of the engine is done entirely in Netlogo language as a Netlogo model. Therefore, the experimenters can easily modify or add pieces of code as required. In order to demonstrate its functionality and usability, the proposed engine has been used to implement an agent representing an experimental artificial insect which learns to navigate in a simulated two-dimensional world avoiding obstacles and predators while searching for food. The agent is controlled by a simple neural circuit which demonstrates some of the SNN neural dynamics including temporal and spatial summation of incoming pulses, spike generation, spike timing dependent plasticity (STDP) learning [19–21] and synaptic delay.

Why Netlogo? Netlogo is a software application that provides an integrated environment for the simulation and programming of multi-agent models and the study of emergent behaviour in complex systems [22]. The Netlogo programming language offers a set of primitives which allows the agents to perceive and modify their virtual world and also to communicate and interact with other agents. In terms of simplicity, as stated by Tisue and Wilensky [22], Netlogo is built on the slogan “low threshold, high ceiling platform” inherited from Logo which describes the language as approachable for students and novices but at the same time providing the capabilities required by advanced users in order to create sophisticated models.

Apart from its simplicity, one of the main advantages of using Netlogo in this work is that it allows to monitor and manipulate on each single simulation iteration the state of each element of the neural circuit including: (1) neurons and their internal variables, (2) synapses and their parameters (efficacy and delay) and (3) ongoing pulses. Manipulation of the neural circuit can be done with commands given through the observer prompt or by using the agent monitoring tool provided by the Netlogo GUI. The architecture of the engine is explained in detail in the following sections.

## Methodology

### The implemented spiking model

The proposed SNN engine is a simplification of integrate and fire (I&F) models which recreate to some extent the phenomenological dynamics of neurons while abstracting the biophysical processes behind them. In the simplest terms, the implemented model assumes that the only inputs come from pulses of presynaptic neurons and without any imposed external currents.

For the simulation, a similar approach to Upegui et al. [23] has been adopted, modelling the artificial neuron as a finite-state machine where the states transitions depend mainly on a variable representing the membrane potential of the cell. All the characteristics of the artificial neuron including: (1) membrane potential, (2) resting potential, (3) spike threshold, (4) excitatory and inhibitory postsynaptic response, (5) exponential decay rate and (6) absolute and refractory periods are enclosed in two possible states: open and absolute refractory.

**Fig. 1:**
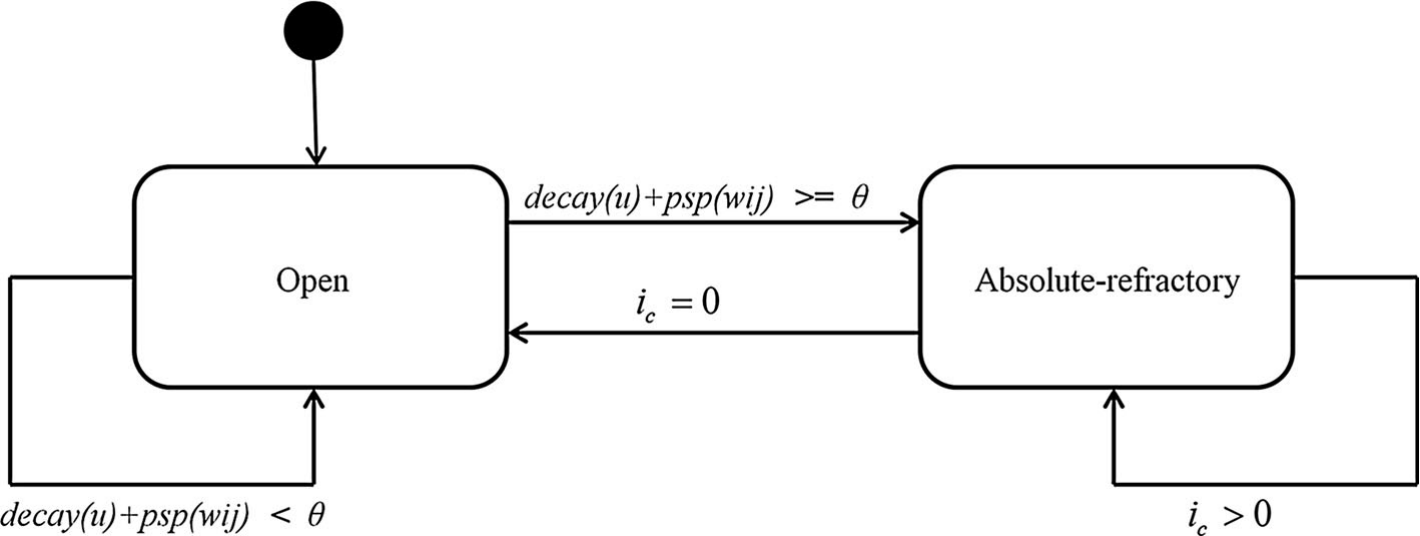
Model state transition represented with a Harel state chart

In the open state, the neuron is receptive to input pulses coming from presynaptic neurons. The amplitude of postsynaptic potentials elicited by presynaptic pulses is given by the function $psp(w_{ij})$ (see Fig. 1) where $w_{ij}$ is the synaptic efficacy between presynaptic neuron *j* and postsynaptic neuron *i*. The membrane perturbations reported by $psp(w_{ij})$ are added (excitatory postsynaptic potential EPSP) or subtracted (inhibitory postsynaptic potential IPSP) to the actual value of the membrane potential *u*.

**Fig. 2:**
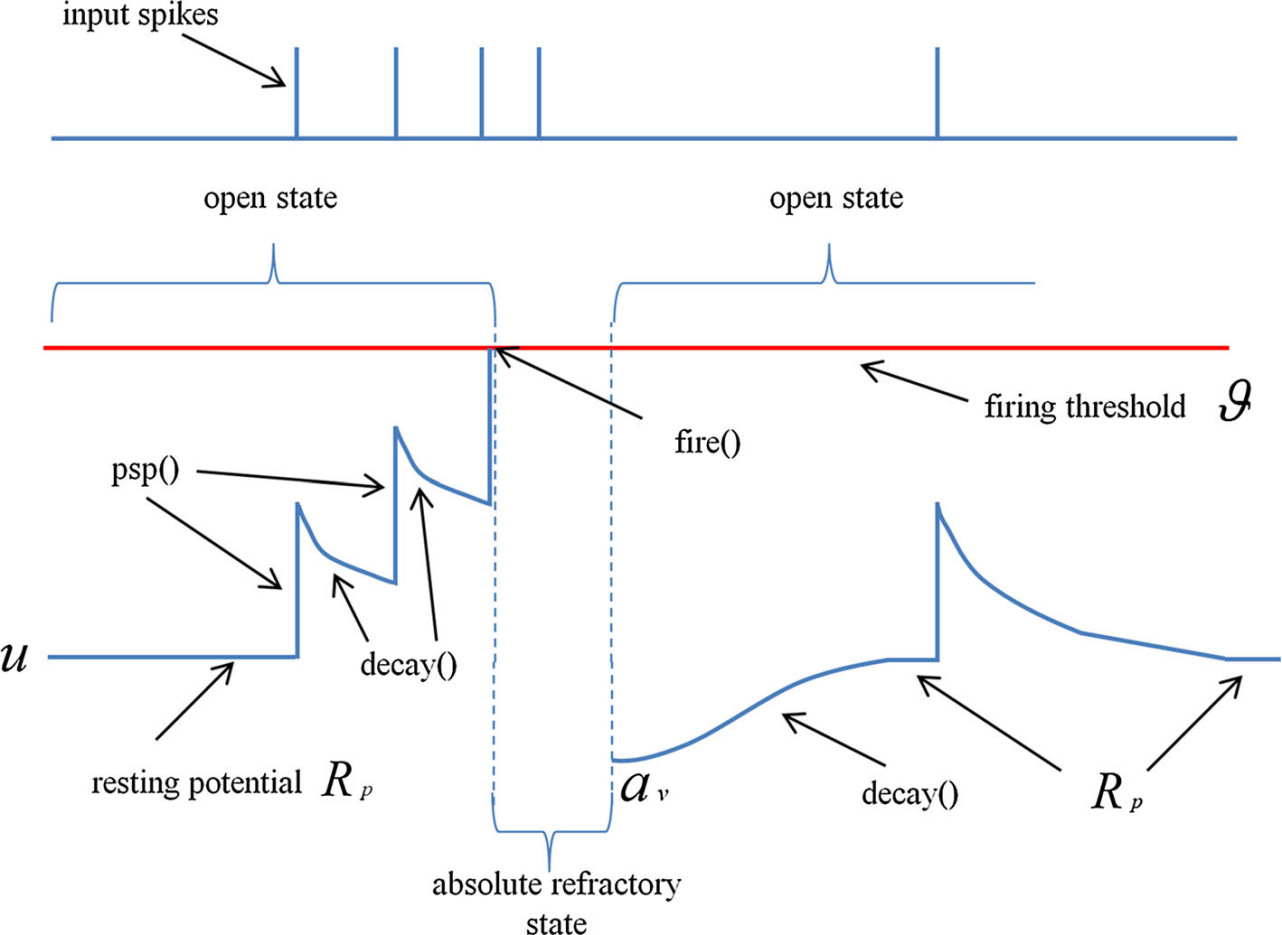
Modelling of the membrane potential in the implemented SNN model

If the neuron firing threshold $\theta$ is not reached by *u*, then *u* begins to decay [see decay(*u*) in Fig. 1] towards a fixed resting potential ${\rm{r}}_p$. On the other hand, as occurs in other integrate and fire implementations, if the membrane potential reaches a set threshold, an action potential or spiking process is initiated. In the presented model, when *u* reaches the firing threshold $\theta$, this triggers a state transition from the open to the absolute-refractory state. During the latter, *u* is set to a fixed refractory potential value $a_v$ (see Fig. 2) and all incoming presynaptic pulses are neglected by *u*. At the initiation of the absolute-refractory state, an iteration counter $i_c$ is set to a value $n_r$ representing the duration of this state expressed in number of simulation iterations (or Netlogo ticks). Once the $n_r$ iterations are completed, a state transition to the open state is triggered by the condition $i_c = 0$.

As shown in Fig. 1 all the dynamics of the simulated neuron are encapsulated within the two states, open and absolute refractory, while the entire states transition depends on the two variables *u* and $i_c$.

Figure 2 below illustrates the behaviour of the membrane potential in response to incoming presynaptic spikes according to the simulation approach explained above:

The following algorithm which is based on one of the simulation methods suggested by Jahnke et al. [24] illustrates how the different processes are implemented within the two machine states:

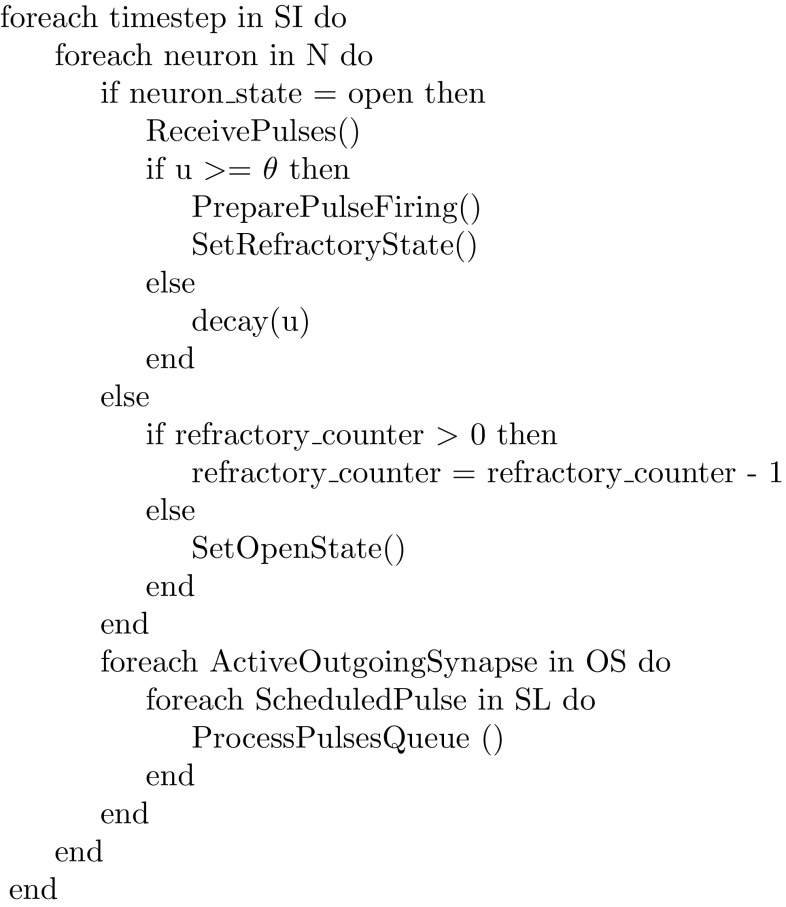



In the algorithm above, SI indicates the number of simulation iterations or time steps. *N* represents the number of neurons each having two operational states determined by the variable neuron_state. In the open state, ReceivePulses() is receiving and handling the incoming pulses. If an action potential is triggered (membrane potential *u* ≥ threshold *θ*), an output spike is generated and transmitted (taking into account synaptic delays) by PreparePulseFiring(). The schedule of outgoing pulses is followed by SetRefractoryState() which performs the state transition to absolute refractory and initializes the refractory_counter with the (fix) duration of the absolute refractory period. If the condition (*u* ≥ *θ*) is not satisfied, *decay*(*u*) brings *u* towards the neuron resting potential on each iteration. If neuron_state is not open, then the state is assumed to be absolute_refractory. In this state, refractory_counter is decreased on each iteration until reaching zero meaning the end of the absolute refractory period and the transition to open state. Independently of the neuron state, the simulation iterates through the list of neuron outgoing-synapses OS. For each outgoing synapse, the pulses waiting to be transmitted SL are processed by ProcessPulsesQueue().

### Comparison with the canonical integrate and fire model

Below we list the main differences and similarities between our implementation and the canonical integrate and fire model.

**Table:** 

	Integrate and fire model (I&F)	Our model
Membrane potential	The canonical integrate and fire [25] represents the evolution of the neurone membrane potential through the time derivative of the Law of Capacitance: $I(t) = C_m {\frac{{\text{d}}V_m(t)}{{\text{d}}t}}$ ‘Integrate’ refers to the behaviour of the model when input currents are applied resulting in the increase of the membrane voltage until it reaches a set threshold which initiates a spike (fire event). The I&F model does not implement the decay of the membrane voltage towards its resting potential. Thus the membrane will keep a sub-threshold voltage indefinitely until new input currents make the membrane cross the firing threshold	The evolution of the membrane potential over time is described by the variable *u*. The behaviour of *u*(*t*) depends on: (1) the machine state at time *t*, (2) the applied currents from incoming spikes and (3) the membrane potential leakiness (see below)
Leakiness	The decay or leakiness of the membrane potential is implemented as an extension of the I&F model: the leaky integrate-and fire Model (LI&F) recreates the dynamics of a neuron by means of a current I flowing through the parallel connection of a resistor with a capacitor in an electrical circuit [17, 19]. The current *I* splits in the resistor *R* and capacitor *C*, as follows: $I(t) = I_R + I_C = \frac{u(t)}{R} + C \frac{{\text{d}}u}{{\text{d}}t}$ where the voltage across the capacitor *C* is depicted with *u* and represents the neuron membrane potential. By introducing the membrane time constant $T_{\text{m}} = RC$, the above equation can be rewritten as: $T_{\text{m}} \frac{du}{dt} = -u(t) + RI(t)$ with $T_{\text{m}}$ quantifying the rate at which *u* decays to its resting potential	The decay of the membrane potential *u* is implemented through the decay() process by using two different functions (negative_leak_kernel and positive_leak_kernel) to describe the hyperpolarization and depolarization processes, respectively: If Rest_pot $< u < \theta$ then $u = u -$ negative_leak_kernel else If $u <$ Rest_pot then $u = u +$ positive_leak_kernel
		Rest_pot is the resting potential and $\theta$ the firing threshold. In our model, both negative and positive kernels implement exponential decay functions
Spike initiation	The mechanism of spike initiation is established through a threshold condition: $u(t) = \theta$. Thus, if a given threshold $\theta$ is reached at $t = t^{(f)} ,$ then the neuron is said to fire a spike at time $t^{(f)}$	Same as I&F Fixed firing threshold
Action potential	The form of the generated action potential is not described explicitly in the LI&F model [17]. Following the fire event, the potential is reset: $u_{\text{reset}}$ $< \theta$. Then, when $t > t^{(f)}$ the dynamic behaviour continues as described by the membrane time constant $T_{\text{m}}$	Same as I&F During the generation of action potential, the system initializes the absolute_refractory_period timer
Refractoriness	The absolute refractory period is generally implemented by temporarily stopping the dynamics immediately after the threshold conditions have been reached. After the stop time, the membrane potential dynamics start again with $u = u_{\text{reset}}$ where $u_{\text{reset}} < \theta$	Same as I&F The state of the system remains as absolute_refractory as long as the absolute_refractory_period timer is still alive
Synapses	Following the framework of the I&F model, given a neuron i, its total input current is defined as the sum of all its incoming current pulses: $T_i(t) = \sum _j w_{ij} \sum _f \alpha \left(t - t^{(f)}_j\right)$ where $\alpha (t-t^{(f)}_j)$ describes the time course from the presynaptic firing time *t*(*f*) at neuron *j* and the arrival time *t* at postsynaptic neuron *i*. $W_{ij}$ represents the synaptic weight or efficacy between neuron *j* and the postsynaptic neuron *i*. The postsynaptic current generated by an incoming spike depends on the elicited change in the conductance of the postsynaptic membrane [19]	Similarly to I&F, the total input current is also expressed as: $T_i(t) = \sum _j w_{ij} \sum _f \alpha (t - t^{(f)}_j)$ However, in contrast with the I&F framework, in our model the postsynaptic current only takes into account the efficacy $W_{ij}$ of the synapses but not the conductance of the postsynaptic membrane

### The virtual brain for the virtual insect

#### The STDP learning rule

In this paper, the STDP model proposed by Gerstner et al. [19] has been implemented and used as the underlying learning mechanism for the proposed experimental neural circuit. In STDP, the synaptic efficacy is adjusted according to the relative timing of the incoming presynaptic spikes and the action potential triggered at the postsynaptic neuron.

This can be expressed as follows:

1. The presynaptic spikes that arrive shortly before (within a given range or learning window) the postsynaptic neuron fires are considered as contributors for the depolarization of the postsynaptic neuron. Consequently, these spikes reinforce the weights (in terms of artificial neurons) of their respective synapses.
2. The presynaptic spikes that arrive shortly after (within a given range or learning window) the postsynaptic neuron fires are not considered as contributors for the action potential of the postsynaptic neuron. Consequently, these spikes weaken the weights of their respective synapses.

The following formula [19] describes the weight change of a synapse through the STDP model for presynaptic and postsynaptic neurons represented with *j* and *i* respectively. The arrival times of the presynaptic spikes at the postsynaptic neuron are indicated by $t^f_j$ where $f = 1, 2, 3, \ldots N$ enumerates the presynaptic spikes. $t^n_i$ with $n = 1, 2, 3,\ldots N$ counts the firing times of the postsynaptic neuron *i*:

1 $$\Delta w_j = \mathop {\sum }\limits ^N_{f=1} \mathop {\sum }\limits ^N_{n=1} W\left(t^n_i - t^f_j\right)$$

Let $\Delta t = t^n_i - t^f_j$ .The connection weight resulting from the combination of a presynaptic spike with a postsynaptic action potential is given by the function [19–21]:

2 $$\begin{aligned} W(\Delta t) = \left\{ \begin{array}{ll} A_+ \exp (-\Delta t / \tau _+), &{}\quad {\hbox { if }} \, \Delta t > 0 \\ -A_- \exp (\Delta t / \tau _-), &{}\quad {\hbox {if}} \, \Delta t < 0 \end{array}\right. \end{aligned}$$

The parameters $A_+$ and $A_-$ indicate the amplitude of the potentiation and depression of the synaptic weights, respectively. $\tau _+$ and $\tau _-$ are the time constants which describe the exponential shape of the learning window.

In order to create a neural circuit of Spiking neurons that allow the association of an innate response with a neutral stimulus, it is necessary to have at least the following elements:

1. A receptor or sensory input for the unconditioned stimulus *U*.
2. A receptor or sensory input for the conditioned or neutral stimulus *C*.
3. The motoneuron or actuator activated by the unconditioned stimulus *M*.

For *U*, the unconditioned stimulus must be able to elicit an immediate reflex-response (action potential) in the postsynaptic motoneuron. Thus the synapse efficacy of the presynaptic neuron *U* (unconditioned input neuron) must be greater or equal to the activation threshold of the motoneuron() in order to elicit a postsynaptic action potential with a single presynaptic spike. For *C,* the conditioned stimulus must be able to elicit a PSP (postsynaptic potential) in the postsynaptic motoneuron *M*. Thus a synapse between the presynaptic neuron *C* (conditioned input neuron) and the postsynaptic motoneuron *M* must exist. Given the elements *U*, *C* and *M*, the following topology could be used to illustrate a simple associative neural circuit:

**Fig. 3:**
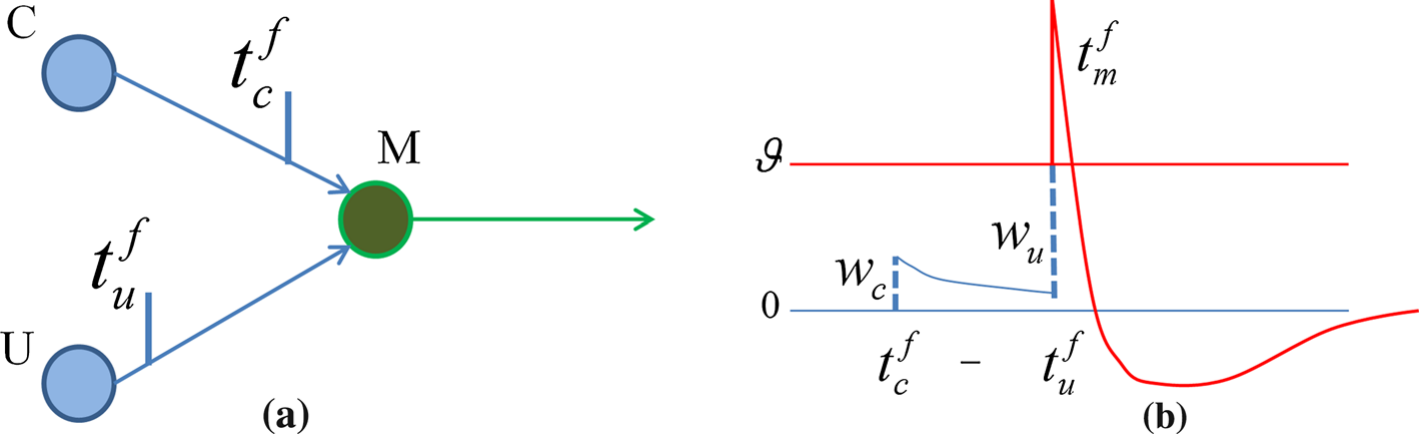
Basic associative topology. **a** Spikes emitted by input neurons *C* and *U* reaching the synapse with postsynaptic motoneuron *M* at times $t^f_c$ and $t^f_u,$ respectively. **b** The* spike* emitted by *C* elicits an EPSP (excitatory postsynaptic potential) of amplitude $w_c$ (*left dashed line*) at time $t^f_c$. At time $t^f_u$ the spike emitted by *U* elicits an EPSP of amplitude $W_u$ (*right dashed line*) that reaches the threshold $\theta$ triggering an action potential at the postsynaptic motoneuron *M*

The neural circuit in Fig. 3 illustrates two input neurons *C* and *U* each transmitting a pulse to postsynaptic neuron *M*. As shown in Fig. 3b, the unconditioned stimulus transmitted by *U* arriving at time $t^f_u$ triggers an action potential in *M* at time $t^f_m$ shortly after the EPSP elicited by *C* at time $t^f_c$.

**Fig. 4:**
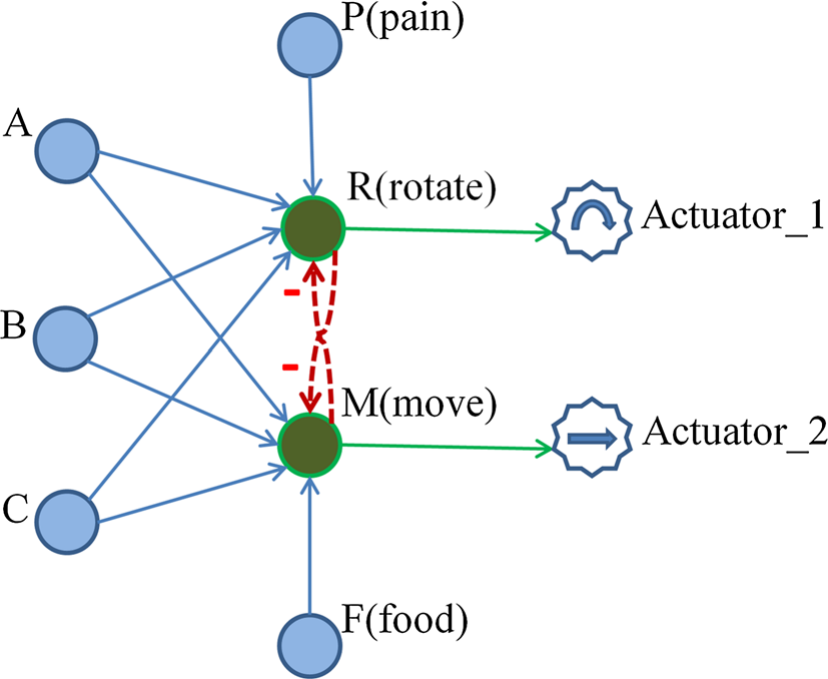
A two-motoneuron circuit

Having $t^f_m > t^f_c$ (EPSP in *M* elicited by *C* preceding spike at *M*) the synaptic efficacy between *C* and *M* ($\Delta w_c$ ) would be increased relative to the difference $t^f_m - t^f_c$ and the set parameters of the STDP learning window [19–21]. This circuit (Fig. 3a) can be taken as a building block to design a simple neural controller working as an artificial micro-brain for the simulated insect. However, on its own, this circuit only allows a limited margin of actions (trigger reflex or not) in response to the input stimuli. The neural topology presented below in Fig. 4 extends the previous circuit in Fig. 3a by adding a second motoneuron and three input neurons:

The topology illustrated in Fig. 4 includes five input neurons *A*, *B*, *C*, *P*, *F* and two motoneurons *R* and *M*. Motoneuron *R* has the function of a negative tropism, which consists in moving away or heading in a different direction (depending on action in actuator_1) when a noxious stimulus is sensed by *P*. In contrast to *R*, motoneuron *M* behaves as a positive tropism, and thus when *F* senses a stimulus, the immediate reaction will be to move towards the direction of the stimulus. Neurons *A*, *B* and *C* receive inputs from three different neutral stimuli. These three neurons are initialized with equal or random synaptic weights with motoneurons *R* and *M*. Neurons *P* and *F* receive their inputs from a pain receptor (nociceptor) and a reward stimulus (e.g. positive-pheromone, food smell, etc.), respectively. The synaptic efficacy between *P* and *R* is defined such that $w_{fm} \ge \theta _m$ in order for the motoneuron *R* to be activated whenever a nociceptive stimulus is received in *P* (reflex reaction of *R* to *P*). Motoneuron *R* triggers Actuator_1 which is depicted with a circular arrow. In a similar way, the synaptic efficacy between *F* and *M* is defined such that in order for the motoneuron *M* to be activated whenever a rewarding stimulus is received in *F* (reflex reaction of *M* to *F*). Motoneuron *M* triggers Actuator_2 depicted with a right-heading arrow. The mutual inhibition between *R* and *M* leads to a winner-takes-all behaviour where the first motoneuron which fires prevents its counterpart to be activated, avoiding the simultaneous activation of both actuators.

### Anatomy of the virtual insect

#### The sensory system

The experimental virtual insect is able to process three types of sensorial information: (1) visual, (2) pain and (3) pleasant or rewarding sensation. The visual information is acquired through three photoreceptors (see Fig. 4) where each one of them is sensitive to one specific colour (black, red or green). Each photoreceptor is connected with one afferent neuron which propagates the input pulses towards the motoneurons. Pain is elicited by a nociceptor whenever the insect collides with a wall or a predator. A rewarding or pleasant sensation is elicited by a pheromone (or nutrient smell) sensor when the insect gets in direct contact with the originating stimulus.

#### The motor system

**Fig. 5:**
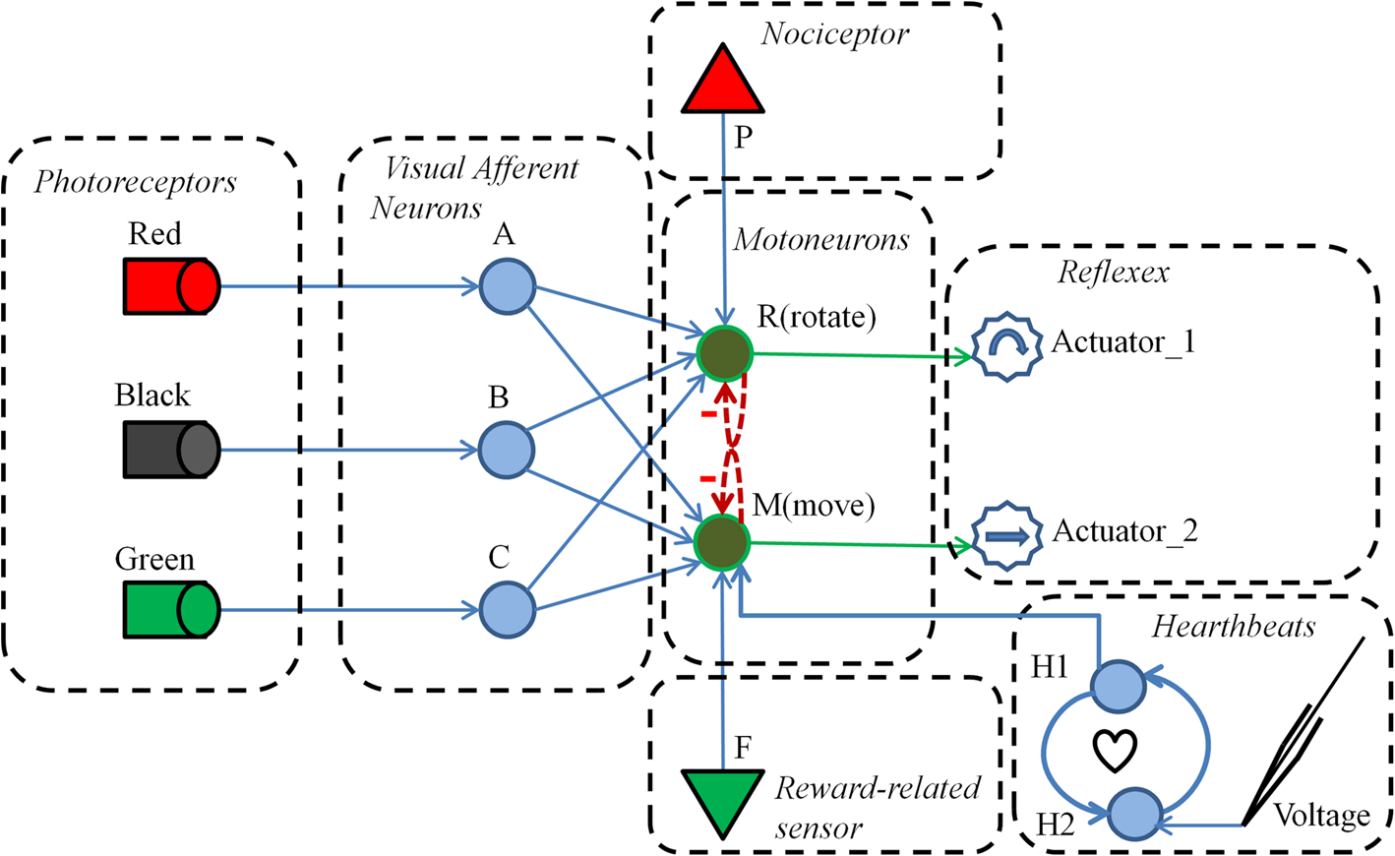
The neural anatomy of the experimental virtual insect

The motor system allows the virtual insect to move forward or rotate in one direction according to the reflexive behaviour described above in Fig. 3. In order to keep the insect moving even in the absence of external stimuli, the motoneuron M is connected to a neural oscillator sub-circuit composed of two neurons *H*1 and *H*2 (see Fig. 5) performing the function of a pacemaker which sends a periodic pulse to *M*. The pacemaker is initiated by a pulse from an input neuron which represents an external input current (i.e; intracellular electrode). Figure 5 below illustrates the complete neural anatomy of the insect.

In Netlogo, there are four types of agents: Turtles, patches, links and the observer [22]. The virtual insect is represented by a turtle agent as well as each neuron in the implemented SNN model. Synapses on the other hand are represented by links. All simulated entities including the insect, Neurons and synapses have their own variables and functions that can be manipulated using standard Netlogo commands. The Netlogo virtual world consists of a two-dimensional grid of patches where each patch corresponds to a point (*x*, *y*) in the plane. In a similar way to the turtles, the patches own a set of primitives which allow the manipulation of their characteristics and also the programming of new functionalities and their interaction with other agents. The visualization of the simulation is divided in two areas inside Netlogo’s world-view interface: (1) The neural circuit topology which is shown on the left half of the screen and (2) the insect and its environment which are shown on the right half side of the screen.

**Fig. 6:**
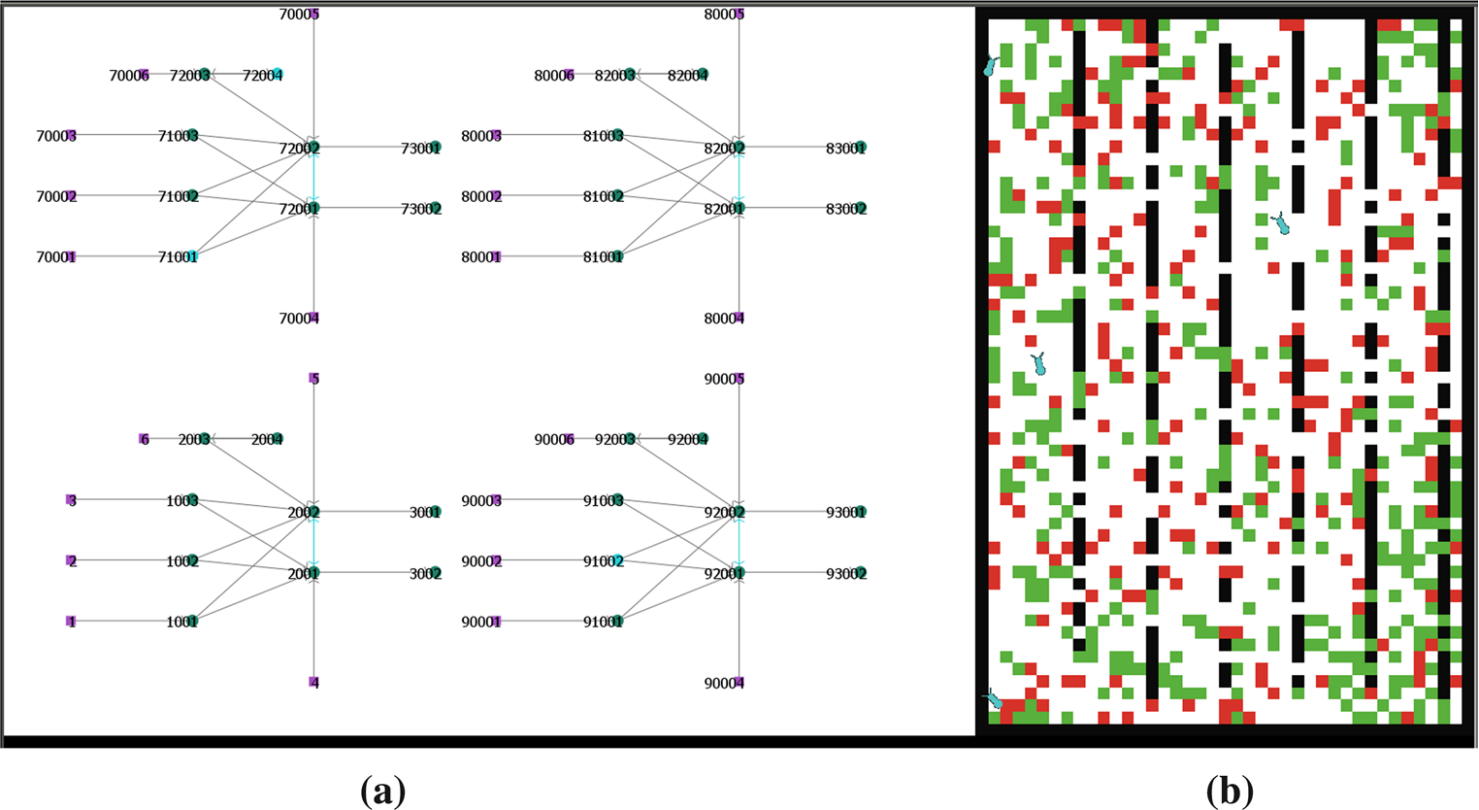
Netlogo’s world-interface. **a** Neural circuits. **b** The simulated insect environment

The topology screen shown in Fig. 6a on the left reflects any change (adding or removing components) done to the neural circuit in each simulation iteration. The world screen in Fig. 6b shows the simulated virtual world including patches of three different colours: black, red and green representing walls, harmful and rewarding stimuli, respectively. The virtual insect is represented with an ant-shaped agent that starts moving once the simulation is initiated. In addition to the simulated world, Netlogo provides different interface objects for plotting and monitoring agents behaviour. In the presented simulation, two plots have been implemented in order to visualize the change over time of the membrane potential of any two neurons selected by the experimenter.

## Results

### The SNN Netlogo-engine

Using Netlogo version 5.2 on a personal computer running Windows 7 with a CPU Intel Core i7 at 2.9 GHz and 8 Gigabytes of RAM, the simulation of the SNN engine (including the proposed neural circuit, two membrane potential plots and the simulated insect’s virtual environment) was able to run at an average of 10,000 iterations or ticks per second (tps) using continuous update mode and with model speed set as fastest in the Netlogo GUI. In order to test the scalability of the engine, four instances of the virtual insect with their corresponding neural circuits were implemented and tested simultaneously within the same Netlogo model (see Fig. 6). Table 1 below summarizes these results:

**Table 1:** Netlogo-ticks/second simulating up to four virtual insects simultaneously

Number of insects	Average ticks per second (tps)
1	10,000
2	6800
3	4000
4	3200

### The virtual insect

**Fig. 7:**
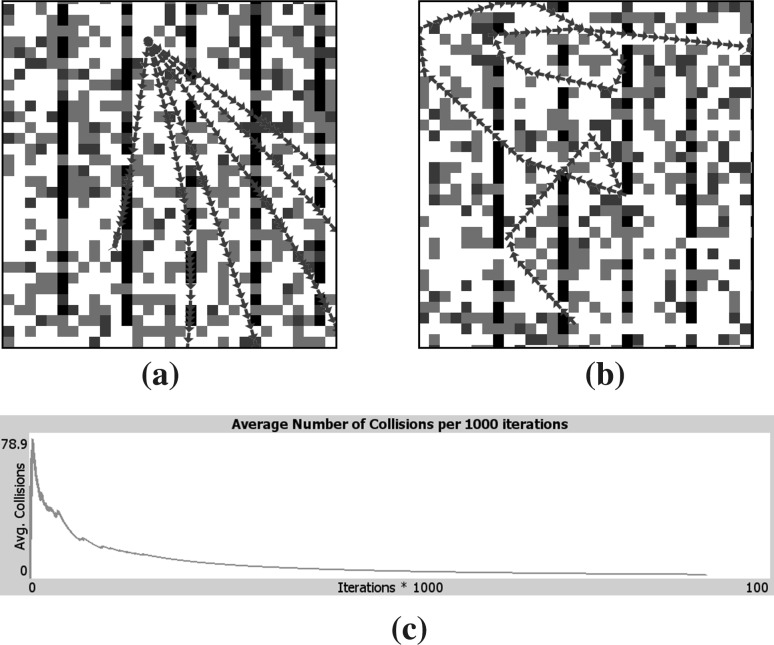
Trajectories and number of collisions during the simulation. **a** Short trajectories at the beginning of the simulation. **b** Long trajectory shows insect avoiding *red* and *black* patches. **c** Number of collisions decreasing as simulation continues

The blue arrows in Fig. 7a and b indicate the different trajectories taken by the virtual insect at different times during the simulation. At the beginning in Fig. 7a, the insect moves along the virtual world in a seemingly random way colliding equally with all types of different objects (coloured patches). During the training phase, the insect is repositioned in its initial coordinates every time it reaches the virtual world boundaries. Figure 7b shows the trajectory between 15 and 25 thousand iterations. It can be seen that the trajectories lengthen as the learning of the insect progresses and more obstacles (walls and harmful stimuli) are avoided. The behaviour in (a) and (b) is reflected by the plot in Fig. 7c which shows the average number of collisions with obstacles (black and red patches) per thousand iterations. The peak at the beginning of the plot shows the highest measured number of collisions in a time slot (of thousand iterations) demonstrating the initial inability of the insect to discriminate and react in response to visual stimuli.

As shown in Fig. 7c, the artificial insect is able to move almost collision free after about 15,000 simulation iterations. However, this number depends on the parameters set for the circuit neural dynamics and the STDP learning rule. Table 2 illustrates the learning behaviour in terms of number of iterations required for a collision-free movement, using different values for the learning constants *A*+ and *A*− (see Eq. 2) to, respectively, potentiate or depress the synaptic weights between the afferent and motoneurons:

**Table 2:** Behaviour with different learning-amplitude parameters *A*+ and *A*−

Symmetric LTP/LTD amplitude change	Number of ticks (iterations) before collision-free movement
0.01	19,000
0.02	15,000
0.03	9000
0.04	7000

Table 2 demonstrates how changing one of the learning equation parameters affects the overall learning behaviour of the simulation. In a similar way, other STDP and neural parameters can be manipulated in the proposed SNN engine in order to experiment with the emergent dynamics.

## Summary and conclusions

This work has presented (1) the implementation of a SNN model in Netlogo and (2) the creation of a neural circuit using the proposed SNN model applied to the control of an agent in a simulated two-dimensional world.

With regard to the implementation of the SNN model, the proposed SNN model was able to run four small neural circuits while demonstrating its ability to reproduce simple but fundamental neural dynamics including: space and time summation of incoming pulses, action potential with refractory period, synaptic plasticity (based on STDP) and synaptic delays. However, the implemented SNN model neglects a substantial part of the features of biological neurons and does not include many of the kernel functions to simulate more complex dynamics as done by other simulation tools. This is expected, since the implemented model is aimed at being an educational tool to introduce SNN dynamics and at providing a SNN engine for fast prototyping of simple neural circuits with small populations of neurons.

During the experiments, the implemented model was able to support the monitoring and manipulation of every single neuron, synapse and pulse variables in both interactive (step by step) and continuous execution. Moreover, the fact that the simulation was able to run at a speed of over thousand iterations per second even with four circuits and agents running simultaneously demonstrated that the proposed model can be used to simulate interactions between multiple agents controlled by spiking neural circuits at a reasonable speed. Still, as shown in Table 1, when the number of neurons and synapses increases, the performance of the simulation drops significantly going from 15 neurons (having 15 neurons per insect) at 10,000 tps to 60 neurons (4 insects) at 3200 tps. Thus, scalability is an issue. This is due to the fact that Netlogo (version 5.2 at the time) runs as a single thread process and thus multiple cores cannot be used to improve the performance of the simulation. An alternative to overcome some of the performance limitations would be using the Netlogo java API extension to implement parts of the model in native java code.

With regard to the application to the control of a virtual agent, the artificial insect experiment demonstrated that an agent controlled by SNN can adapt to its environment by means of associative learning using STDP. The results in Table 2 showed that different learning rates can be achieved by manipulating the STDP equation parameters.

The presented model can be further developed by modifying or implementing new kernels in order to extended the biological characteristics and to support more complex dynamics. However, any further development will have to take into account the imposed limitations in terms of performance and scalability.

## Appendix

See Table 3.

**Table 3:** Parameters used in the implemented spiking neuron model and the STDP learning rule

Parameters	Neurons A, B, C, R, M, H1, H2, Actuator_1 and Actuator_2
Resting potential	−65
Firing threshold	−55
Decay rate amplitude	0.5
Refractory potential	−75 (−70 for neurons H1 and H2)
Duration of absolute refractory state	1
Weight increase amplitude $A_+$	0.09
Weight decrease amplitude $A_-$	−0.09
Highest weight limit	9
Lowest weight limit	1
Positive learning window interval	55
Negative learning window interval	−25
Learning window potentiation time constant $\tau _+$	8
Learning window depression time constant $\tau _-$	15
